# The Synergistic Role of Irreversible Electroporation and Chemotherapy for Locally Advanced Pancreatic Cancer

**DOI:** 10.3389/fonc.2022.843769

**Published:** 2022-05-25

**Authors:** Argyrios Gyftopoulos, Ioannis A. Ziogas, Andrew S. Barbas, Dimitrios Moris

**Affiliations:** ^1^ Medical School, National and Kapodistrian University of Athens, Athens, Greece; ^2^ Department of Surgery, Division of Hepatobiliary Surgery and Liver Transplantation, Vanderbilt University Medical Center, Nashville, TN, United States; ^3^ Department of Surgery, Duke University Medical Center, Durham, NC, United States

**Keywords:** irreversible electroporation, locally advanced pancreatic cancer, chemotherapy, electrochemotherapy, electrogene, immunotherapy

## Abstract

Irreversible electroporation (IRE) is a local ablative technique used in conjunction with chemotherapy to treat locally advanced pancreatic cancer (LAPC). The combination of IRE and chemotherapy has showed increased overall survival when compared to chemotherapy alone, pointing towards a possible facilitating effect of IRE on chemotherapeutic drug action and delivery. This review aims to present current chemotherapeutic regimens for LAPC and their co-implementation with IRE, with an emphasis on possible molecular augmentative mechanisms of drug delivery and action. Moreover, the potentiating mechanism of IRE on immunotherapy, M1 oncolytic virus and dendritic cell (DC)-based treatments is briefly explored. Investigating the synergistic effect of IRE on currently established treatment regimens as well as newer ones, may present exciting new possibilities for future studies seeking to improve current LAPC treatment algorithms.

## Introduction

By 2030, pancreatic cancer is expected to be the second most common cause of malignancy-related mortality in the United States (1). Although surgical resection is the mainstay treatment with curative intent, only up to 30% of pancreatic cancer cases are amenable to resection (2, 3). Hence, according to the extent of vascular involvement and tumor resectability, non-metastatic disease is classified as resectable, borderline resectable, and locally advanced pancreatic cancer (LAPC) (4), with LAPC having a variable definition depending on the different consensus guidelines (**Table 1**) (5–11). The standard of care for resectable pancreatic cancer is resection followed by adjuvant chemotherapy, and for borderline pancreatic cancer is neoadjuvant therapy (12). A recent meta-analysis of randomized clinical trials also demonstrated that neoadjuvant gemcitabine-based chemo(radio)therapy – without adjuvant FOLFIRINOX (5-fluorouracil/leucovorin plus oxaliplatin and irinotecan) – led to improved overall survival in patients with borderline resectable pancreatic cancer, yet its role in resectable cases warrants further exploration (4). The introduction of more effective chemotherapy regimens, such as FOLFIRINOX and gemcitabine plus albumin-bound paclitaxel, within the past 10 years, has also been important for patients with LAPC as both the resectability and survival of LAPC after neoadjuvant therapy have increased (13–20). Therefore, either FOLFIRINOX or gemcitabine plus albumin-bound paclitaxel chemotherapy is currently the first-line treatment for patients with LAPC, and response is monitored using biochemical (e.g., carbohydrate antigen 19-9), radiographic [e.g., Response Evaluation Criteria in Solid Tumors (RECIST) criteria on computed tomography scan], and metabolic response (e.g., positron emission tomography scan) criteria (21).

**Table 1:** Locally advanced pancreatic cancer definition according to different guidelines (5).

Vascular anatomy	NCCN (6)	AHPBA/SSO/SSAT (7)	MDACC (8)	Alliance (9)	ISGPS (10)	IAP (11)
**Lesion at head or uncinate process of the pancreas**						
**SMA/Portal vein**	Involvement not amenable to reconstruction	Occlusion not amenable to reconstruction OR venous encasement with encasement of nearby arteries	Unreconstructable segmental occlusion without normal vein proximally and/or distally	Occlusion not amenable to reconstruction	Involvement with distortion/narrowing and/or occlusion not amenable to reconstruction	Bilateral narrowing/ occlusion, exceeding the inferior border of the duodenum
**SMA**	Tumor involvement >180°	Tumor involvement >180°	Tumor involvement >180° OR dense tissue involving the vessel	Tumor-vessel interface >180°	Tumor contact >180°	Tumor contact >180°
**CHA**	Long-segment encasement or any celiac abutment OR IVC or aortic invasion	Long-segment encasement OR extension to celiac axis	Long-segment encasement not readily reconstructable	Unreconstructable tumor-vessel interface (long-segment, abnormal adjacent vessel)	Long-segment encasement or celiac axis involvement	Tumor contact of proper hepatic artery and/or celiac axis
**Lesion at body or tail of the pancreas**						
**Celiac axis**	Tumor contact >180° OR aortic invasion	Abutment/encasement	Tumor contact >180°	Tumor-vessel interface >180 °degrees	Tumor contact >180° OR aortic invasion	Abutment >180° OR any aortic or GDA involvement

AHPBA, Americas Hepato-Pancreato-Biliary Association; Alliance, Alliance for Clinical Trials in Oncology; CHA, common hepatic artery; GDA, gastroduodenal artery; IAP, International Association of Pancreatology; ISGPS, International Study Group of Pancreatic Surgery; IVC, inferior vena cava; MDACC, MD Anderson Cancer Center; NCCN, National Comprehensive Cancer Network; SMA, superior mesenteric artery; SSAT, Society for Surgery of the Alimentary Tract; SSO, Society of Surgical Oncology.

Admittedly, the management of patients with LAPC unresponsive to neoadjuvant therapy is an area of great debate among the pancreatic surgery community. Of note, the current level of evidence precludes us from deducing meaningful conclusions on whether surgery can yield a survival benefit over a non-surgical approach, especially since these pancreatic resections involve major vessel resections and reconstructions commonly accompanied by great morbidity (22–29). Instead, irreversible electroporation (IRE), a form of nonthermal injury initially used in 2009 for LAPC (30, 31), has been more frequently utilized as a consolidative therapy for LAPC with favorable outcomes. Whereas other locally ablative techniques, such as radiofrequency ablation, have been employed in treating simple-shaped, mass-forming LAPC, IRE is known to target tumors with intricate formations, even in case of major blood vessel encasement (32). In addition, IRE has been proposed to have a complementary role to other modalities, such as chemotherapy, newer immunotherapies, or electrogene therapy. The aim of this review is to discuss current chemotherapy options for LAPC patients and elaborate on the facilitative effect of IRE on intra-tumoral delivery and action of chemotherapy, immunotherapy, newer electrogene and dendritic cell (DC)-based *ex vivo* techniques.

## Current Chemotherapy Options for Locally Advanced Pancreatic Cancer

In the era of genome-wide association studies and next generation sequencing, chemotherapy is tailored to address individual tumors down to the level of single-nucleotide-polymorphisms (33). It has been made possible to estimate qualities, such as tumor burden and resistance to chemotherapeutic regimens, by recognizing key mutations in the cancer cell genome. Nowadays, personalized medicine has evolved into a tool of paramount importance in choosing the appropriate chemotherapeutic regimen against LAPC.

The randomized phase III PRODIGE trial evaluated the use of FOLFIRINOX versus gemcitabine for metastatic pancreatic cancer in patients with good performance status and demonstrated significant improvements in survival using FOLFIRINOX (13). Based on these results, FOLFIRINOX is included as a preferred, category 1 recommendation for first-line therapy for patients with metastatic pancreatic cancer and good performance status [i.e., Eastern Cooperative Oncology Group (ECOG) performance status 0-1]. By extrapolation, this regimen is considered a category 2A recommendation for LAPC patients in the National Comprehensive Cancer Network (NCCN) guidelines (12). However, there are certain concerns regarding FOLFIRINOX-induced toxicity, as in the PRODIGE trial, the FOLFIRINOX group had significantly higher rates of neutropenia, diarrhea, thrombocytopenia, and sensory neuropathy and decreased quality of life compared to the gemcitabine group (13). Therefore, prospective data showed that a modified FOLFIRINOX regimen with a 25% reduced initial bolus dose of 5-fluorouracil and irinotecan can mitigate chemotherapy-induced toxicities without a negative effect on survival; as such, the modified FOLFIRINOX regimen is also included as a preferred treatment option in the NCCN guidelines (12, 34).

For metastatic or LAPC, gemcitabine has shown a modest clinical and survival benefit over bolus 5-fluorouracil (35). Therefore, the NCCN guidelines recommend gemcitabine monotherapy as a category 1 front-line option for good performance status metastatic and LAPC patients and as a reasonable category 1 first- or second-line option for symptomatic poor performance status patients (12). Gemcitabine monotherapy is also a category 1 therapy for adjuvant treatment after resection as the phase III CONKO-001 trial showed a survival benefit using adjuvant gemcitabine over observation after macroscopically complete resection in patients without prior chemotherapy or radiation (36, 37). Additionally, the randomized phase III MPACT trial compared gemcitabine plus albumin-bound paclitaxel versus gemcitabine alone in patients with metastatic pancreatic cancer and no prior chemotherapy and demonstrated improved response and survival in the gemcitabine plus albumin-bound paclitaxel group (14, 38). Therefore, gemcitabine plus albumin-bound paclitaxel is listed in the NCCN guidelines as a preferred category 1 recommendation for good performance status (i.e., ECOG performance status 0-2) metastatic pancreatic cancer patients, and by extrapolation as a category 2A recommendation for LAPC patients as well (12).

## Current Experience With IRE

IRE initially showed promise when applied to animal models (39, 40). Moreover, in human patients, since 2009, Nanoknife (Angiodynamics, Latham, NY, USA) has been used as a Food and Drug Administration-approved IRE delivery system. Currently, IRE has proven to be a safe procedure with a clear benefit in overall survival of LAPC patients when combined with chemotherapy. Recently, experience from a cohort of 40 patients undergoing IRE in Greece from 2015 to 2019 showed a median overall survival of 24.2 months with few major grade III complications (two out of 40 patients developing pancreatic fistula). Importantly, 33 out of 40 patients had undergone FOLFIRINOX or nab-paclitaxel neoadjuvant therapy prior to IRE, and after repeat imaging showed no disease progression, they underwent local ablation through electroporation (41).

Martin et al. (30) initially commented on the safety of IRE in treating LAPC in 2012. They recruited 27 patients with grade III pancreatic adenocarcinoma who had previously received various chemotherapy regimens. Preoperatively, 24 patients had reported 100% performance status. IRE delivery had a 100% technical success. Median stay in the hospital following IRE was 9 days. The cost of the procedure, however, was high (2,000$ per probe). Importantly, the role of extensive surgeon experience using IRE (50 cases at minimum using NanoKnife), or similar ablative techniques, in pancreas or other organs (i.e., liver, kidney), was emphasized, based on predictive learning curves (30).

More recently, in a systematic review by Moris et al. (42) gathering results up to August 2018, IRE was found to be technically feasible and with few side-effects in a total of 498 patients. Open, laparoscopic, and percutaneous approaches were compared. However, results regarding the survival benefit of laparoscopic IRE vs open therapies were ambiguous. Additionally, with open-approach IRE, 35.8% of patients experienced postoperative morbidity, with 21.5% being major incidents (Clavien-Dindo grade ≥III) (42). On the other hand, laparoscopic IRE was associated with lower morbidity (24.3% overall morbidity and 13.3% Clavien-Dindo grade ≥III) (42). The overall reported mortality following IRE was 2% (42). However, open IRE is most commonly performed, as it allows for more accurate placement of the probes and gives the surgeon the opportunity to evaluate whether a LAPC may be resectable, despite previous imaging studies suggesting otherwise (43, 44).

Lafranceschina et al. (45) summarized the outcomes of 691 patients with unresectable LAPC previously treated with chemotherapy who underwent IRE. The CROSSFIRE trial, gathering 138 patients from the Netherlands, and the AHPBA trial, with 500 patients from the USA, Japan, Taiwan, and the UK, as well as smaller studies from China, France, and Canada, were combined. Median patient age was 63 years, and tumor size ranged between 2.8 to 4.5 cm. Ideally, tumor size between 3-4 cm showed best results following IRE. The overall morbidity rate was 30.5% and complications included pancreatic fistula, pancreatitis, thrombosis, and pseudoaneurysm formation, while mortality following IRE was 3.4%. Interestingly, overall survival was between 10-27 months following IRE compared to 6-11.5 months in patients treated with chemotherapy and/or radiation without IRE.

In 2020, Ruarus et al. (46) published a phase II study with a total of 50 patients. Despite an increased survival in patients undergoing IRE compared to chemotherapy, there was no proof of synergy between the two. Ten patients had recurrent LAPC. Overall survival was 17 months following diagnosis (10 months following IRE application), and 16 months in the recurrence group. Only 22 patients had received induction chemotherapy with FOLFIRINOX. Of the 50 patients, there was one recorded death related to IRE. Of note, there was no difference in survival in patients who had received FOLFIRINOX induction vs those with gemcitabine or no chemotherapy. Therefore, IRE was the main determinant of increased survival in those patients. The aim of 12-14 months survival following only conventional chemotherapy was exceeded by patients participating in the study.

A poorly addressed topic in the literature is that of IRE-related morbidity and mortality. In a review of the literature, Charalambous et al. in 2020 included a total of 460 patients across 9 studies (47). They concluded that overall mortality was 3.4% (amongst the causes were peritonitis, hepatojejunostomy site ulceration and bleeding, bile duct necrosis, enterocutaneous fistula, gastroduodenal artery bleeding and multisystem organ failure) while major (Clavien-Dindo class III or higher) morbidity was 10.2% (**Table 2**). Overall morbidity was 29.4% at 90 days following open IRE. An open approach seems to be associated with higher morbidity. Nevertheless, it is the most commonly administered method, and, according to the authors, is associated with increased overall survival versus laparoscopic or percutaneous approaches. Sugumar et al. in 2021 conducted a systematic review of the literature including a total of 2,768 patients (53). They reported 12-month major complication and mortality rates of 18% and 2.65%, respectively, following a combination of multimodal therapy and IRE. One-year overall and progression-free survival rates were 55% and 12%, respectively (53). Importantly, the authors concluded that multimodal LAPC therapy with IRE had a similar overall mortality rate to multimodal therapy without IRE. Therefore, they recommended the use of IRE only as an experimental treatment modality in current clinical practice (53).

**Table 2:** Mortality causes (in patients treated with IRE for LAPC) (45).

Author	Causes	Number of patients affected
Flank (41)	- Purulent peritonitis - Malignant ascites*	2
Holland (48)	- Ulceration at the hepaticojejunostomy, submucosal hematoma with ischemic changes - SSI*	2
Vogel (49)	- Duodenal hemorrhage due to tumor infiltration* - PV thrombosis*	2
Kluger (50)	- Duodenal necrosis - Bile duct necrosis - Deep SSI* - Duodenal-cutaneous fistula - PV thrombosis - GDA hemorrhage	5
Paiella (51)	- Ulcerative colitis flare/Septic shock*	1
Martin (52)	- Duodenal ulcer bleeding* - Pulmonary embolism* - Portal vein thrombosis/Liver failure*	3

*Not directly caused by IRE, according to authors.

DA, Gastroduodenal Artery; IRE, Irreversible Electroporation; LAPC, Locally Advanced Pancreatic Cancer; PV, Portal Vein; SSI, Surgical Site Infection.

## IRE Amplification of LAPC-Targeted Drug Effect and Delivery

**Figure 1:**
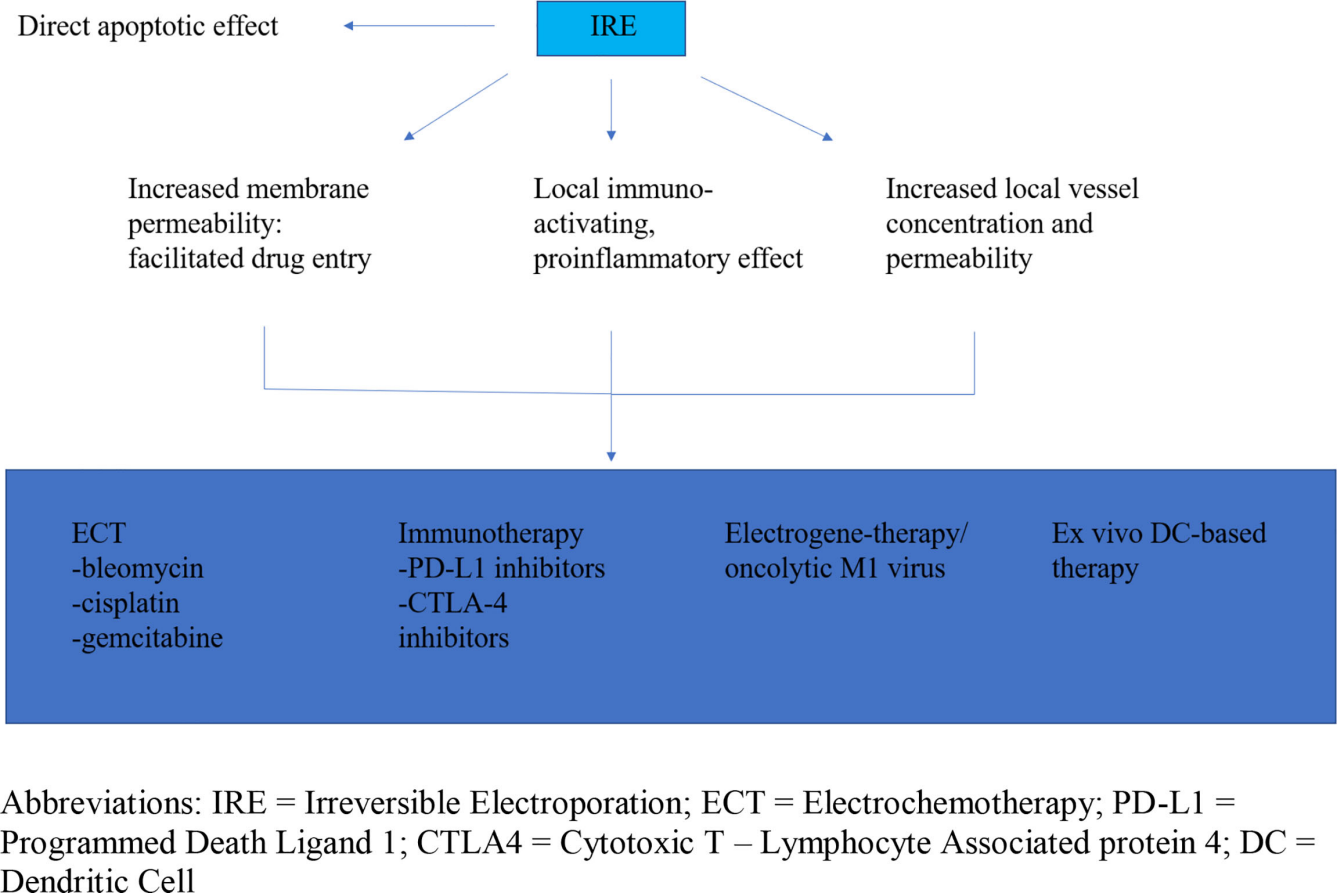
Irreversible electroporation anti-tumor effect and facilitation of drug mechanism and delivery. IRE, Irreversible Electroporation; ECT, Electrochemotherapy; PD-L1, Programmed Death Ligand 1; CTLA4, Cytotoxic T – Lymphocyte Associated protein 4; DC, Dendritic Cell.

Aside from direct apoptotic and possible chemotherapy-augmenting effects, IRE has been postulated to have a local immunologic impact on the tumor bed, which provides an opportunity for immunotherapeutic regimens to take effect (54). Hence, there are many ways IRE can affect tumor cells, either directly by promoting apoptosis, or indirectly, through facilitation of other drug mechanisms (**Figure 1**).

Electrochemotherapy (ECT) is the co-implementation of IRE and chemotherapy in treating LAPC (55). IRE penetrates cell membranes rendering cancer cells susceptible to the effects of chemotherapeutic drugs with otherwise little ability to infiltrate the cell. Current ECT regimens include bleomycin and cisplatin. With regards to pancreatic cancer, few small studies so far have exhibited promising results with minimal side effects (56–58). Most recently, a randomized control trial by Izzo et al. evaluated the effectiveness of ECT with subsequent chemotherapy (FOLFOXIRI) versus only chemotherapy in inoperable LAPC and highlighted the importance of multiple insertions with variable geometry ensuring a more complete coverage of the tumoral lesion as this can lead to improvements in both local disease control and overall survival (59). However, one drawback of ECT is its limited effect on distant metastases, despite its good local suppressive effects (60). Recently, IRE-facilitated intra-tumoral transfer of gemcitabine, an established chemotherapeutic regimen for metastatic pancreatic cancer and patients with poor functional status, has also demonstrated promising results (61). Finally, another study beginning in 2021 aims to compare overall survival and progression-free survival rates of ECT (bleomycin) to IRE and calcium electroporation (i.e., the influx of calcium in electro-porated cells resulting in cell death) in pancreatic cancer patients with poor prognosis in Poland (62). It remains to be seen whether those innovative approaches to dealing with inoperable disease will provide a viable alternative for LAPC patients.

Newer immunotherapeutic regimens, such as immune checkpoint inhibitors against cytotoxic T-lymphocyte-associated protein 4 and programmed cell death protein-1, did not show favorable results when used alone for LAPC (63). Notably, IRE is known to have an immunologic effect, affecting the tumor milieu in such a way that it tips the scale from local immunosuppression to inflammation and tumor cell immune recognition and destruction (64). Namely, 14 days after IRE application, there was notable helper and memory T cell number proliferation, whilst Tregs were shown to be decreased. The number of macrophages rose as well, while natural killer (NK) cells did not show a significant increase (65–67). Therefore, it would be clinically interesting to examine the interaction of IRE with current immunotherapy regimens during this short window of immunologic opportunity.

Electrogene-therapy is the transfer of therapeutic genes inside tumor cells by means of electroporation (60). A similar principle is used in M1 virus insertion into pancreatic cancer cells. M1 is an anti-tumor RNA virus whose protein product stimulates cancer cell apoptosis. M1 oncolytic virus-treated mice with LAPC showed increased survival after treatment (68). Following IRE, pores are created on the cell surface, allowing M1 to enter without requiring a specialized viral transporter. Furthermore, the local application of IRE enhances vessel permeability and increases local vessel concentration within the tumor bed, thereby achieving larger concentrations of M1 virus. And as IRE transforms the immune-suppressed tumor microenvironment into an immuno-active, proinflammatory one, T cell activation against M1 oncolytic virus-infected cells is facilitated even further.

DCs are antigen-presenting cells of the immune system with a role in stimulating immune response against foreign antigens. DCs presenting certain antigens on their surface are taken from patients and cultured *ex vivo*. They are then re-introduced into the patient, exerting an anti-tumor effect by alerting the host immune system. Local tumor immunosuppression, however, has an inhibitory effect on the action of DCs on the tumor bed (69, 70). Once again, the local immune-activating effect of IRE allows downstream activated T and B cells to penetrate cancer cells, therefore facilitating the effect of injected DCs (71).

## Application of IRE in Conjunction With Chemotherapy

**Figure 2:**
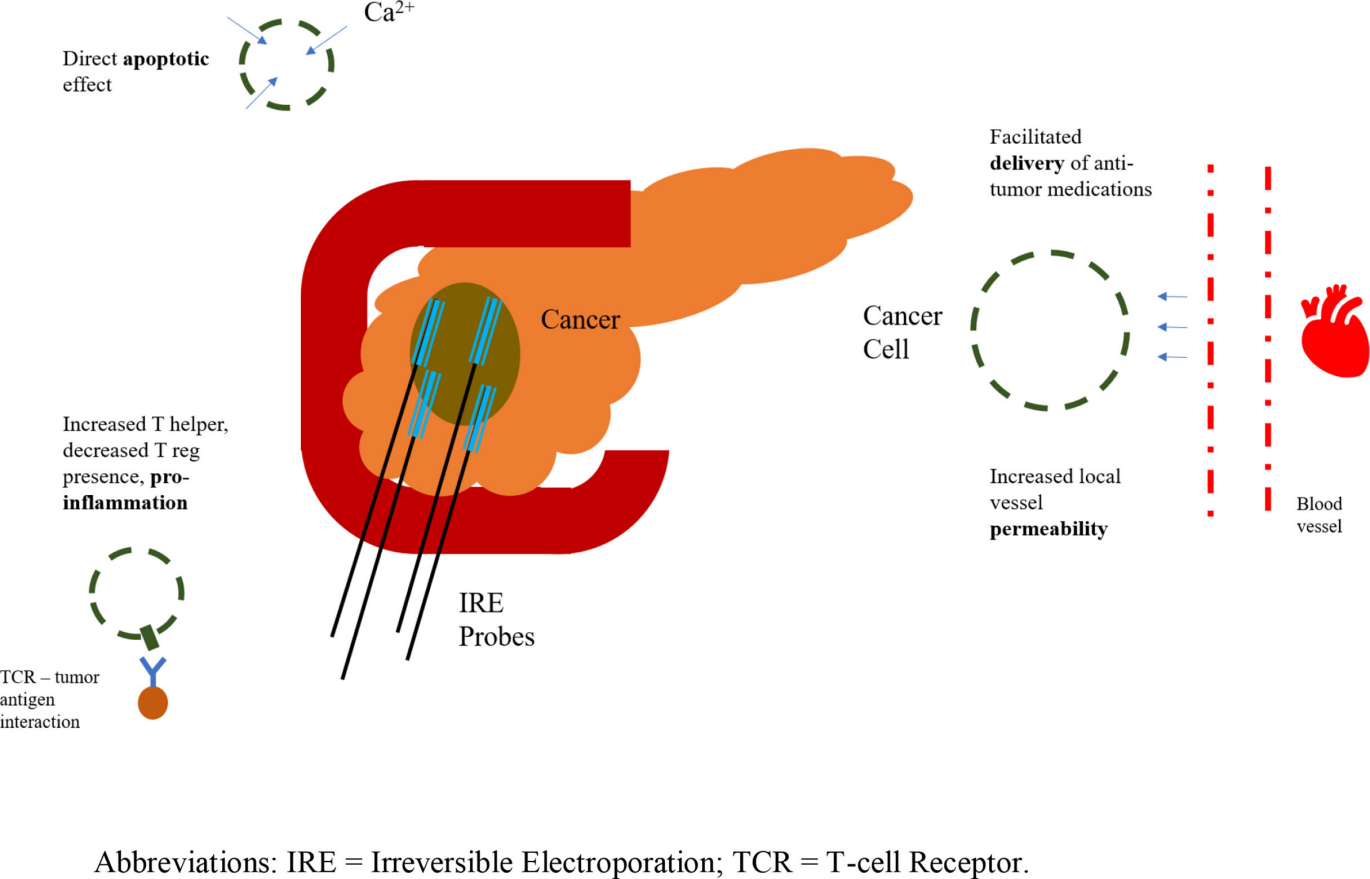
Irreversible electroporation application; representation of probes and anti-tumor effects. IRE, Irreversible Electroporation; TCR, T-cell Receptor.

Before commencing with either FOLFIRINOX or gemcitabine and subsequent IRE, triphasic computed tomography scan with pancreatic protocol (0.7 mm cuts) and three-dimensional reconstruction is performed to appropriately stage the tumor (72). The presence or absence of superior mesenteric artery or vein or celiac artery encasement, distant metastases or peritoneal spread on imaging will guide the choice of chemotherapy and determine whether an operative approach is possible (5–11). Moreover, diagnostic laparoscopy is performed with paracolic and pelvic washing to detect smaller distant tumor foci that would again signify inoperable disease. Importantly, IRE is a locally ablative technique with no effect on distant metastases. After induction chemotherapy, and if no metastases are detected on repeat imaging, and pancreatic tumor axial diameter is < 4.0 cm, IRE can be performed 2-4 weeks after the last dose of chemotherapy, per RECIST criteria (**Figure 2**) (73).

Chemotherapy in LAPC aims to shrink the existing tumor to make negative-margin surgical resection feasible. Neoadjuvant chemotherapy has also decreased the need for vascular reconstruction, a particularly challenging aspect of surgical resection for LAPC (74). In the past, vascular reconstruction was carried out at highly specialized centers but was not a technique widely available (75, 76). However, advances in surgical technique combined with newer chemotherapeutic regimens have rendered vascular resection and subsequent reconstruction in LAPC a common practice (77). Currently, patients with LAPC being treated with chemotherapy can either remain stable or, rarely, have their disease downgraded (78). In a subset of patients, unfortunately, LAPC can progress and metastasize during neoadjuvant chemotherapy. Should LAPC remain stable or be downgraded to borderline resectable disease, surgical excision becomes an option (75). Locally ablative techniques, such as IRE, can also be implemented towards that goal. Indeed, Sadot et al. in 2015 reported that in a total of 101 patients with LAPC and median follow-up of 12 months, following 6 cycles of FOLFIRINOX, one third were able to undergo local resection (25). Suker et al. in 2016 conducted a systematic review showing that close to 30 percent of patients receiving FOLFIRINOX were able to have their LAPC resected by the end of the treatment (91 of 325 patients) (79). However, if metastases occur, the tumor is inoperable and there is no utility in performing IRE, since it has no effect on distant foci of disease. Sadot et al. reported that 23% of their patients developed distant metastatic foci by the end of the neoadjuvant treatment (25). Hence, the benefit of possible synergism between chemotherapy and IRE must be weighed against the risk of disease progression during initial neoadjuvant chemotherapy.

An important consideration regarding current chemotherapy regimens (i.e., FOLFIRINOX, gemcitabine) is that trials to date have primarily included patients with metastatic disease, not LAPC (13, 14). While chemotherapy can be used in both metastatic and LAPC, IRE is only applicable in the latter. LAPC-specific randomized clinical trials that compare FOLFIRINOX to gemcitabine are yet to be undertaken.

The mechanism by which IRE facilitates the delivery and action of chemotherapeutic drugs is complex. Chemotherapy, however, even without IRE, has an established benefit in treating pancreatic cancer patients. While studies have shown mixed results, the application of neoadjuvant chemotherapy with subsequent IRE has previously proven superior to chemotherapy alone in extending overall survival in patients with LAPC (80). This possible synergism points to an potentiating effect of IRE on existing chemotherapeutic regimen mechanism of action. Understanding the way IRE facilitates the delivery and action of various immunotherapeutic regimens, electrogene modalities and DC based treatments is key in incorporating IRE in the current standard treatment algorithm for LAPC, including chemotherapy and possibly surgical resection.

## Conclusion

IRE has a direct pro-apoptotic effect on LAPC cells by increasing membrane permeability and disrupting cancer cell homeostasis. However, it also seems to facilitate the delivery and action of chemotherapeutic regimens (bleomycin, cisplatin, and gemcitabine). Indeed, adjunctive chemotherapy followed by IRE has shown superior overall survival over chemotherapy alone. IRE, however, also appears to augment the effect of immunotherapy, M1 oncovirus- and DC-based therapies for LAPC. Further research is needed to examine the potentiating effects of IRE on anti-LAPC drug delivery and action. Current examination of potential facilitating mechanisms points to a key role of IRE in future LAPC treatment algorithms.

## Author Contributions

AG, IZ, and DM conceived and designed the study, acquired, analyzed, and interpreted the data, drafted, and critically revised the manuscript, and approved the final version of the manuscript. All authors contributed to the article and approved the submitted version.

## Conflict of Interest

The authors declare that the research was conducted in the absence of any commercial or financial relationships that could be construed as a potential conflict of interest.

## Publisher’s Note

All claims expressed in this article are solely those of the authors and do not necessarily represent those of their affiliated organizations, or those of the publisher, the editors and the reviewers. Any product that may be evaluated in this article, or claim that may be made by its manufacturer, is not guaranteed or endorsed by the publisher.

## Abbreviations

IRE, Irreversible Electroporation; LAPC, Locally Advanced Pancreatic Cancer, ECT, Electrochemotherapy, DC, Dendritic Cell.
